# Acute fibrinous and organizing pneumonia presents as right lung upper lobe cavitary lesion: Case report and literature review

**DOI:** 10.1097/MD.0000000000044915

**Published:** 2025-09-26

**Authors:** Cui-Cui Wang, Yan Chen, Meng-Jie Li, Biao Xie, Qin Shan, Hanlong Guo, Qu-Cheng Huang, Bang-Ming Guo

**Affiliations:** a Department of Oncology, Renmin Hospital of Qingxian, Cangzhou, China; b Department of Anesthesiology, General Hospital of Central Theater Command of the People’s Liberation Army, Wuhan, China; c Department of Pulmonary and Critical Care Medicine, Renmin Hospital of Qingxian, Cangzhou, China; d Department of Thoracic Cardiovascular Surgery, General Hospital of Central Theater Command of the People’s Liberation Army, Wuhan, China; e Shandong University of Traditional Chinese Medicine, Jinan, China; f Department of The First Clinical Medical College, Gannan Medical University, Ganzhou, China; g Department of Neurosurgery, First Affiliated Hospital with Gannan Medical University, Ganzhou, China.

**Keywords:** acute fibrinous and organizing pneumonia, case report, cavitary lesions, cystic lung diseases, lung cancer

## Abstract

**Rationale:**

The upper lobe of the right lung, due to its unique anatomical structure that is prone to tumor occurrence, poses a challenge for the differential diagnosis of focal cavitary lesions. Cases of acute fibrinous and organizing pneumonia presenting as cavitary lesions in the upper lobe of the right lung are extremely rare in the previous literature.

**Patient concerns:**

This case presents a 59-year-old man who was hospitalized with a cough, phlegm, and low-grade fever after a bout of strenuous exercise and exposure to rain. A chest computed tomography (CT) revealed a cavitary lesion (lesions maximum diameter 9 cm, hollow maximum diameter of 3.5 cm) in the upper lobe of the right lung.

**Diagnoses:**

Initially, he was incorrectly diagnosed with community-acquired pneumonia, and the empirical anti-infective and antiviral therapies proved ineffective. The flexible bronchoscopy lavage fluid showed negative results in metagenomic Next-Generation Sequencing (mNGS), but the pathological micrograph indicated a suspected diagnosis of acute fibrinous and organizing pneumonia.

**Interventions:**

He was taken off antibiotics, changed to 40 mg of methylprednisolone intravenously each day.

**Outcomes:**

Six days after receiving methylprednisolone, the cough disappeared. A reexamination chest CT showed a significant reduction of the lesion. Additionally, the patient did not report any discomfort during the 6 months of follow-up.

**Lessons:**

This case highlights the particular characteristics of the focal cavitary lesions in the upper lobe of the right lung and emphasizes the role of liquid-based cytology in the diagnosis of acute fibrinous and organizing pneumonia, rather than mNGS.

## 1. Introduction

Lung tumorous lesions are most common in the upper lobe of the right lung, and pulmonary tuberculosis is also common in the upper lobe.^[1,2]^ Pulmonary abscesses are most common in the right lung, and are most common in the anterior, posterior, and inferior lobes of the upper right lung.^[3,4]^ Lung tumorous, pulmonary tuberculosis and pulmonary abscess were the main causes of lung cavity lesions.^[5]^ Therefore, lung cavity lesions occurring in the upper lobe of the right lung are the most difficult to accurately diagnose based on patient history, clinical symptoms, and imaging findings. Although metagenomic next-generation sequencing (mNGS) has achieved extremely high sensitivity and specificity in the diagnosis of infectious diseases (bacterial or viral) and neoplastic lesions,^[6,7]^ there are exceptions in clinical practice. This case report aims to describe the exception diagnosis with a right upper lobe lung cavity lesion, which was liquid-based cytology suspected diagnosed as acute fibrinous and organizing pneumonia (AFOP) by cytopathology.

## 2. Case report

A 59-year-old male patient was hospitalized with cough, phlegm, and low-grade fever. These symptoms occurred after a bout of strenuous exercise and exposure to rain. Before hospitalization, they had persisted for 1 week. The sputum is brown, seemingly bloodshot, about 20 mL/d. He also had a low fever with a maximum temperature of 38.0°C. In the past 3 days, he had fatigue, night sweats, poor eating, no chest pain, no hemoptysis, no nausea, no vomiting, no abdominal pain, no diarrhea, no convulsions and no disturbance of consciousness. Based on his disease history, he incorrectly diagnosed with CAP, and received symptomatic empirical treatment, which included tooth and oral cleaning and empirical anti-infective and antiviral therapies (moxifloxacin, 500 mg each day and Oseltamivir Capsules, 75 milligrams each time, twice a day). However, the patient had no obvious relief of the above symptoms and developed. He has a smoking history of 20 years, 20 cigarettes/day, has never quit smoking, and has no drinking history. He had not traveled recently. He had no pets, and had no history of recent dental procedures. He has a history of diabetes for more than 20 years, and is currently taking tablets treatment (metformin, 0.25 g once, 3 times a day; Acarbose, 50 mg once, 3 times daily). Random blood glucose was controlled at 10 to 15 mmol/L after medication. He suffered from hypertension for more than 10 years also, up to 180/100 mm Hg, and is currently treated with oral Irbesartan tablets, 150 mg daily. The patient’s normal blood pressure was controlled at 130 to 140 / 80 to 95 mm Hg. He denied the history of coronary heart disease, food and drug allergies. He was not diagnosed outside the hospital and came to see a doctor because the above symptoms did not improve. Physical examination showed body temperature as 37.6°C, pulse rate as 76 times/min; respiratory rate as 20 times/min, blood pressure as 144/81 mm Hg. The patient was found to be conscious, and his breathing was stable, superficial lymph nodes were not large, the bi-lateral lung percussion was clear, the left lung breathing sound was clear, and the upper right lung was audible with moist rales. He was found to have a heart rate of 76 beats/min, rhythmical consistency, no murmur, flat and soft abdomen, no tenderness in the whole abdomen, no rebound pain and muscle tension, no touch of the liver, spleen and ribs, no edema and varicose veins in both lower limbs.

**Figure 1.**
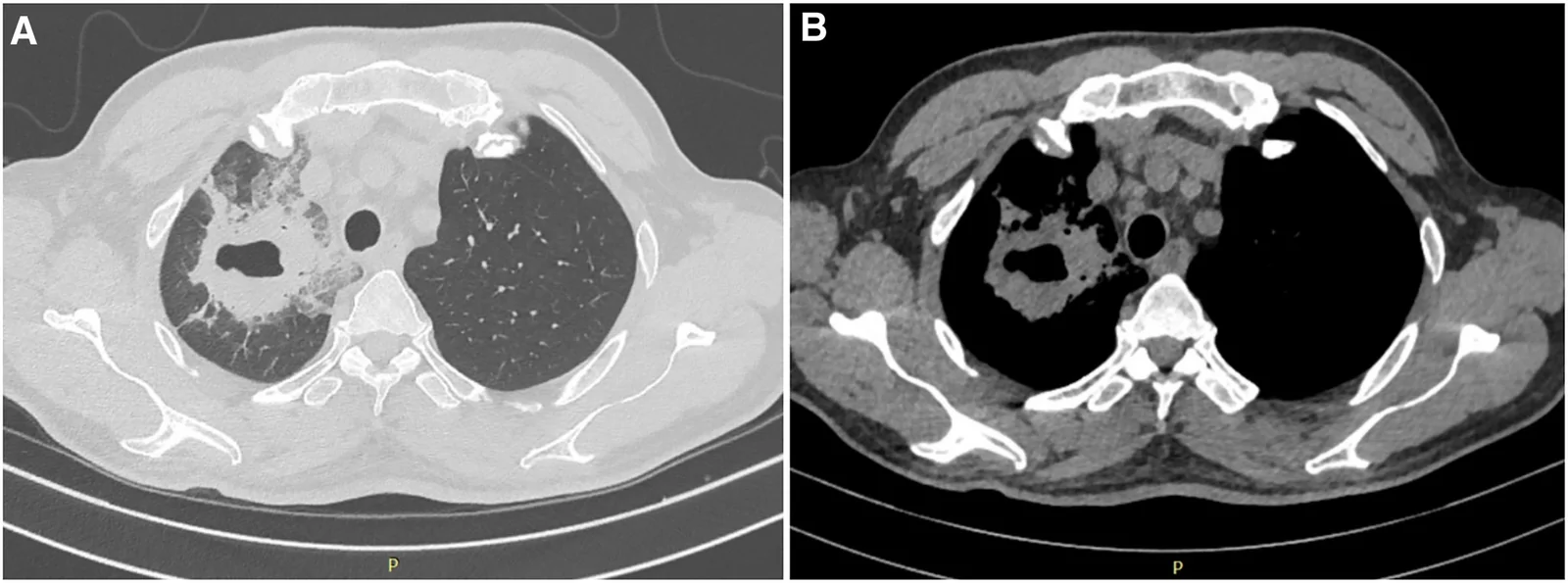
16-slice computed tomographic scan revealed that a cavity lesion in the upper lobe of the right lung, the lesion size was about 9 × 6 cm and the cavity size was about 3.5 × 2.5 cm. (A) lung window; (B) mediation window.

His CT examination revealed a cavity lesion in the upper tip segment of the right lung with a diameter of about 9 cm (lesions maximum diameter 9 cm, hollow maximum diameter of 3.5 cm, Fig. 1A and B). The wall of the cavity is thick, and no gas-liquid plane can be seen inside. Around the irregular, visible wall nodule can be found. The outer margin of the cavity ap-pears to be lobed, with obvious burrs. There were no obvious enlarged lymph nodes in the mediastinum and left hilum. The radiologist thinks it’s probably neoplastic. And the artificial intelligence system (supported by YITU Healthcare Technology Co., Ltd., Hangzhou, China) judged as high-risk nodules of lung cancer.

**Figure 2.**
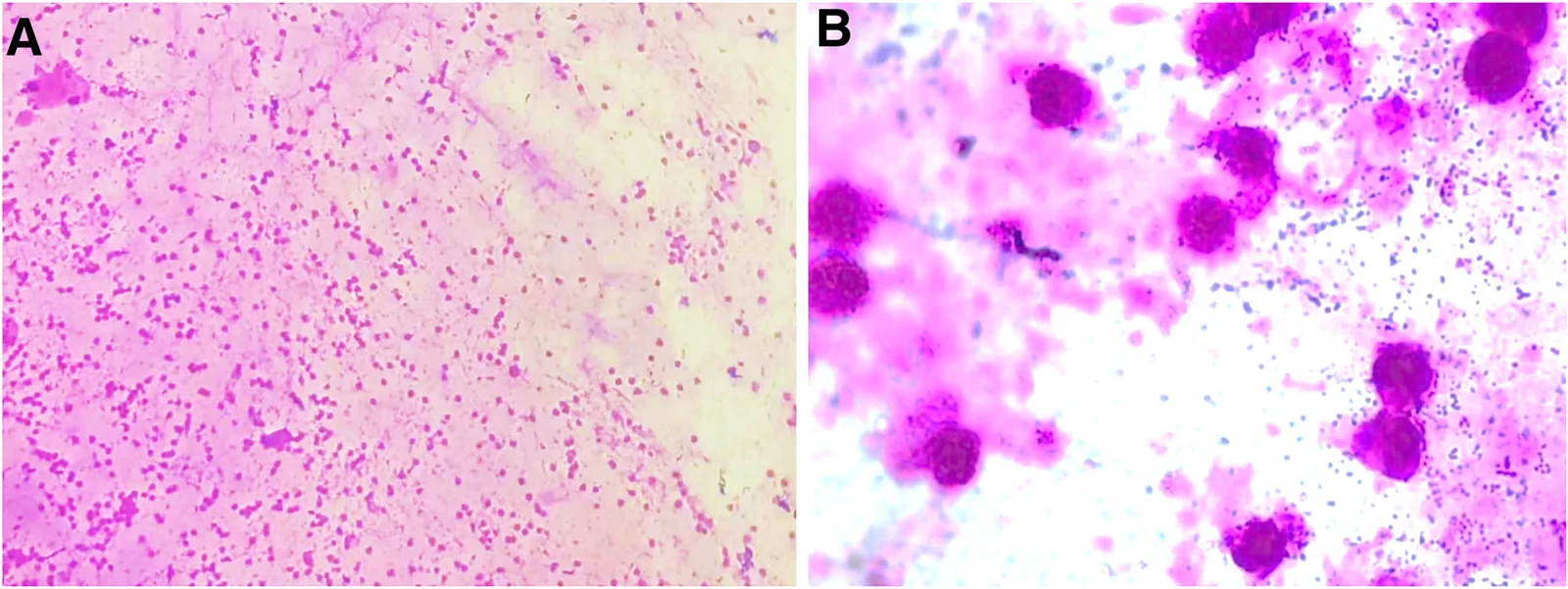
Results of liquid-based cytology by fiberoptic bronchoscopic lavage fluid. (A) 40×; (B) 100×.

Laboratory tests showed routine blood: WBC 11.1 × 10^9^/L (normal range: 3.5–9.5 × 10^9^/L), N% 81.7% (normal range: 40–75), L%1.29 × 10^9^/L (normal range: 1.1–3.2), E%0.02 × 10^9^/L (normal range: 0.02–0.52), CRP 4 mg/L (normal range: 0–8). PCT 0.05 ng/mL (normal range: <0.5). On room air arterial blood gas, PH was 7.49, PCO_2_ was 33.2 mm Hg, PO_2_ was 68.7 mm Hg. Liver and renal function, autoimmune antibody tests, T-SPOT, Erythrocyte sedimentation rate, ANCA detection, fungal G test and GM test, HIV, as well as molecular assay for tuberculosis were otherwise unremarkable. Serum tumor markers, such as CEA, NSE, and CA-129, were normal. Positive results were not found from sputum and blood culture repeatedly. There were no abnormal findings in the airway lumen by bronchofiberscopy, and no positive findings on mNGS of the lavage fluid analysis. Liquid-based cytology of the lavage fluid analysis found macrophages, focal loose noncaseating granulomas, and lymphocytes. No acid-fast or fungal organisms were identified on the slides (Fig. 2A and B). Referring to the patient’s disease progression, the pathological micrograph suggested a diagnosis of AFOP.

**Figure 3.**
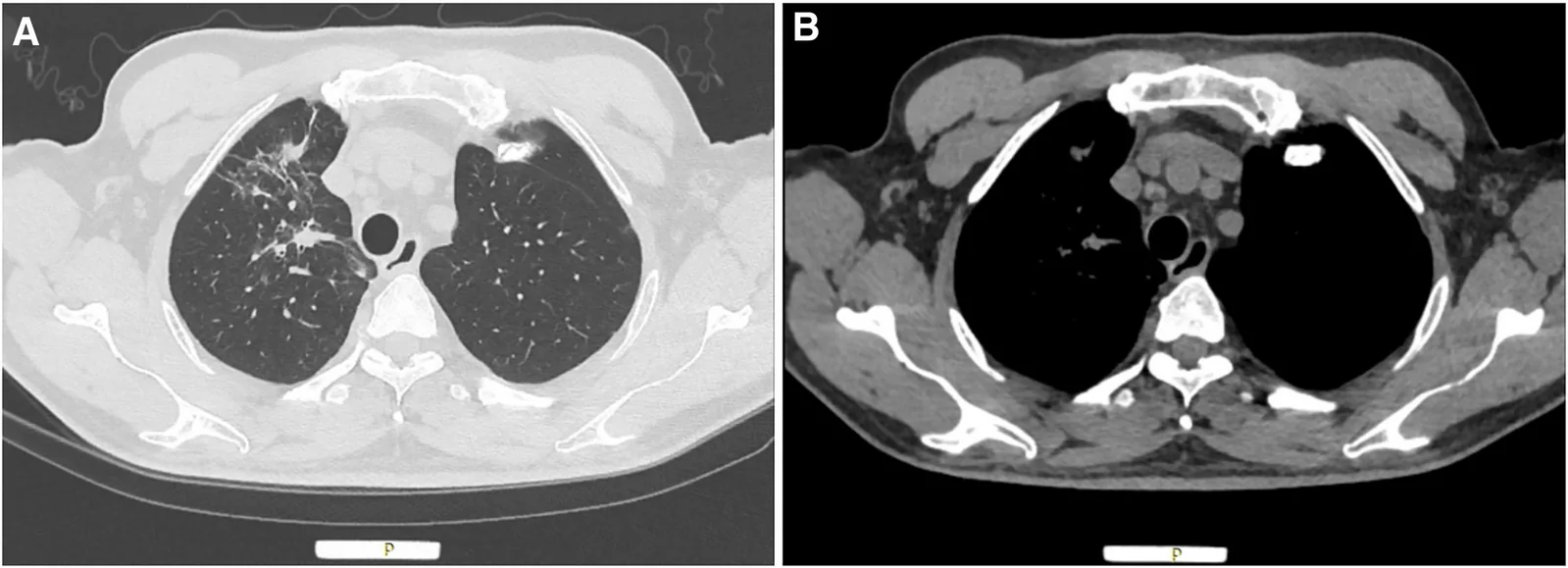
After 6 d of methylprednisolone treatment, 16-slice computed tomographic scan showed that the right upper lung lesion was significantly smaller and the cavity had disappeared. (A) Lung window; (B) mediation window.

According to this suspected diagnosis, the discussion by the multidisciplinary team (MDT) discontinued the antibiotic. The adopted treatment was 40 mg methylprednisolone daily was given intravenously. After 6 days, the cough disappeared. Reexamination chest CT significant reduction of the lesion (Fig. 3A and B). Then, the patient was sent home with a prescription of 24 mg of methylprednisolone orally per day, which was cut down to 16 mg per day after a week. At the 6-month checkup, the patient had no symptoms and experienced no apparent negative reactions to the steroids. So, the patient was very satisfied with the current treatment, which reduced the procedures of the examination and avoided invasive needle biopsies/surgical procedures.

## 3. Discussion

The lesion of the upper lobe of the right lung has always been a difficult diagnostic problem in the departments of respiratory medicine, infection, thoracic surgery and oncology. This is closely related to the anatomical structure of the upper lobe of the right lung, and the bronchial anatomy of the upper lobe of the right lung is the shortest way to approach the respiratory tract and communicate with the air.^[8,9]^ In addition, the main bronchus of the right lung is thicker and easier to inhale foreign bodies such as pollen and germs.^[10]^ Many diseases occur in the upper lobe of the right lung, and the common ones are lung cancer, tuberculosis, lung abscess, and even the incidence of COVID-19 is not low.^[11]^

Lung cavitary lesions are often caused by inflammatory, infectious, or neoplastic processes in contrast to cystic lung lesion.^[12]^ Cystic lung lesion refers to abnormal expansion of physiological spaces in the lung, the wall is thinner than the cavity wall, generally about 1 mm.^[13]^ Cystic lung lesions are found in congenital lung cysts and bulla. Staphylococcus aureus caused air sacs also be-long to cystic lung lesions.^[13,14]^ These lesions were single or multiple. In terms of lesion location, congenital lung cysts can occur in any part of the lung, and pulmonary bulla usually occurs in the lung apex.^[14,15]^ Most cases in the cavity are only gas, when combined with infection can appear liquid flat.^[13,15,16]^

Lung cavity lesions are necrotic lesions in the lung, and necrotic tissue is discharged through the drainage bronchus to form a cavity.^[17]^ Lung cavity lesions can be seen in tuberculosis, lung abscess, lung cancer, mycosis and granuloma.^[5,17]^ Among them tuberculosis, lung abscess and lung cancer are more common.^[5]^ When the necrotic tissue in the cavity cannot be completely discharged through the drainage bronchus, the liquefied necrotic tissue can form a liquid plane in the cavity, which is a common sign of abscess.^[5,17]^

Tuberculosis also presents as cavity lesion. It is characterized by wormlike cavities, also known as warless cavities, often multiple, most commonly seen in cheese pneumonia.^[18]^ Tuberculous thick-walled voids are mostly fibrous caseous voids, with many airless liquid planes inside. Calcification can be seen in the wall of the voids, and satellite and fiber strip foci are often sur-rounding the voids, with tuberculous foci on the same side and/or opposite side.^[18,19]^

Most of lung cavity lesions in lung cancer are eccentric, uneven in thickness, uneven in the inner margin with wall nodules, predisposed to leaf shape, often visible burr, and may be accompanied by ipsilateral hilar and mediastinal lymph node enlargement.^[20,21]^ In this case, the lung cavity had no fluid and was flat inside, while the cavity had uneven inner margin with wall nodules, and the outer cavity was foliated with burrs, so the artificial intelligence mistakenly diagnosed it as a lung tumor lesion.

In addition, some clinical symptoms are also important clues to the diagnosis of lung cavity lesions. Coughing up blood is more conducive to the diagnosis of lung cancer, and fever is more conducive to the diagnosis of pulmonary infectious diseases.^[13,22]^ The bias of the cavity also help diagnosis, which was not conducive to the diagnosis of lung squamous cell carcinoma when the cavity was subpleural. And eccentric cavity is beneficial to the diagnosis of lung squamous cell carcinoma.^[23,24]^ Clear blood vessels can be seen through the cavity, which is not conducive to the diagnosis of lung cancer.^[24]^ There are halo signs around the lesion, which is conducive to the diagnosis of infectious lesions.^[13,17]^ Isolated nodules or spherical lesions in the cavity are seen in pulmonary tuberculosis, pulmonary abscess, residual necrosis or associated with mold spheres.^[13,17]^ The inner surface of the cave wall is uneven, and sometimes nodules can be seen on the inner wall, which is called wall nodules, which is a sign of lung tumor lesions.^[21,23]^

The CT findings of patients with cavity pulmonary tuberculosis may be associated with an empty cavity draining bronchus to the pulmonary hilum.^[18]^ Lung cancer cavity lesions can cause proximal bronchial stenosis and obstruction.^[22,23]^ There are satellite foci around tuberculosis, which are spotty or strip-like, and some voids can see strip-like shadows, which are radial and connected with voids, but their distribution is uneven.^[18,19]^ The cavity lesions of lung cancer would have obstructive pneumonia, and the inflammatory images were mostly distributed in the distal hilus of the lung.^[22,23]^

Only few potential causes of cavitary lesions are AFOP, like this case. AFOP is a rare and distinct form of interstitial lung disease characterized by the presence of intra-alveolar fibrin deposition, often referred to as “fibrin balls,” and organizing pneumonia.^[25]^ Unlike other forms of pneumonia, AFOP does not typically present with eosinophils or hyaline membranes, which are common in diffuse alveolar damage. Cavitary lesions in the lungs are unusual in AFOP but have been documented in few cases. A previous literature showed CT imaging revealed peripheral thick-walled cavitary lesion in the left upper lobe, which was initially nondiagnostic until a CT-guided lung biopsy confirmed AFOP.^[25]^ These few cases were notable for the patient’s positive response to steroid treatment, highlighting the variability in therapeutic approaches for AFOP.^[26]^ These cases underscore the importance of considering AFOP in the differential diagnosis of cavitary lung lesions, particularly when initial treatments for common infections are ineffective. Accurate diagnosis often requires pathological confirmation, not the mNGS.^[27,28]^

## 4. Limitations

This was a case report study, which was not highlight the long follow-up period, and lack of histologic diagnosis, diagnosis have limitations.

## 5. Conclusions

In conclusion, we report a case of a difficult to diagnose cavity lesion in the upper lobe of the right lung. This case highlights the particularity of the focal cavitary lesions in the upper lobe of the right lung and emphasizes the role of liquid-based cytology in the diagnosis of AFOP, not the mNGS.

## Author contributions

**Data curation:** Cui-Cui Wang, Yan Chen, Meng-Jie Li, Qin Shan

**Funding acquisition:** Cui-Cui Wang, Bang-Ming Guo

**Methodology:** Cui-Cui Wang, Yan Chen, Bang-Ming Guo

**Writing – original draft:** Cui-Cui Wang

**Investigation:** Yan Chen

**Project administration:** Yan Chen, Bang-Ming Guo

**Writing – review & editing:** Yan Chen, Bang-Ming Guo

**Resources:** Biao Xie, Hanlong Guo, Bang-Ming Guo

**Software:** Biao Xie, Hanlong Guo, Qu-Cheng Huang, Bang-Ming Guo

**Supervision:** Biao Xie, Qin Shan, Qu-Cheng Huang, Bang-Ming Guo

**Validation:** Biao Xie, Qu-Cheng Huang

**Formal analysis:** Hanlong Guo

**Conceptualization:** Bang-Ming Guo
